# Laparoscopic Central Pancreatectomy With Pancreaticogastrostomy: Our Initial Experience

**DOI:** 10.7759/cureus.24542

**Published:** 2022-04-27

**Authors:** Anzor Kvashilava, Badri Kobalava, Giorgi Giorgobiani

**Affiliations:** 1 General Surgery, Aversi Clinic, Tbilisi, GEO; 2 Surgery Department #3, Faculty of Medicine, Tbilisi State Medical University, Tbilsi, GEO; 3 Surgery Department #3, Faculty of Medicine, Tbilisi State Medial University, Tbilisi, GEO

**Keywords:** pancreatic neoplasms, pancreatectomy, surgery, laparoscopy, cystadenoma

## Abstract

Pancreatic parenchyma-preserving procedures performed for benign and low-grade malignant tumors of the neck or body of this organ significantly reduce the incidence of postoperative exocrine and endocrine insufficiency compared to distal pancreatectomy. Tumor enucleation spares pancreatic parenchyma, but it can have positive surgical margins, and postoperative leakage after it is significant.

We present our initial successful experience of laparoscopic central pancreatectomy. A patient was operated on for cystadenoma of the pancreatic neck. The organ was transected proximally with a linear stapler but distally with ultrasonic shares, and a caudal stump was used for the creation of the pancreaticogastrostomy. The postoperative period was uneventful. The four-month follow-up did not reveal any exocrine or endocrine insufficiency.

## Introduction

Benign lesions of the pancreatic neck and body are mostly treated with distal pancreatectomy because of the good radicality and technical simplicity of both open and laparoscopic approaches. Unfortunately, this method has a drawback - the necessity to excise healthy pancreatic tissue. Postoperatively, the deficiency of the parenchyma would cause exocrine and endocrine pancreatic insufficiency.

Enucleation is another treatment option for such tumors. This method usually is not difficult to perform. However, besides less radicality, it usually gets complicated with pancreatic fistula because of the unavoidable parenchyma damage.

Central pancreatectomy covers the weak points of both methods. It preserves pancreatic tissue, preventing parenchyma insufficiency. Also, this method prevents leakage because it does not leave pancreatic ducts exposed. Anastomosis creation is quite challenging. This stage demands the high dexterity of the surgeon, especially using the laparoscopic technique.

## Case presentation

A 35-year-old Caucasian male complained of discomfort and painful attacks in the epigastric area. Three years earlier, an MRI revealed a 10 × 11 mm cystadenoma in the pancreatic body, and surveillance was advised. A year later, the cyst was enlarged up to 12 × 17 mm. Two months before surgery, pain episodes became daily and much worse; therefore, surgery was planned. The patient had a history of SARS-CoV-2 infection of moderate severity four months prior to the operation. The only significant comorbidity was first-class obesity (BMI: 32.3 kg/m²). Preoperative workup results were in the normal range.

After initiating the balanced narcosis combined with thoracic epidural anesthesia, the patient was placed supine in a lithotomy position. The surgeon was standing between the legs of the patient, and the first assistant and the scrub nurse worked from the right and the left side of the patient, respectively.

**Figure 1:**
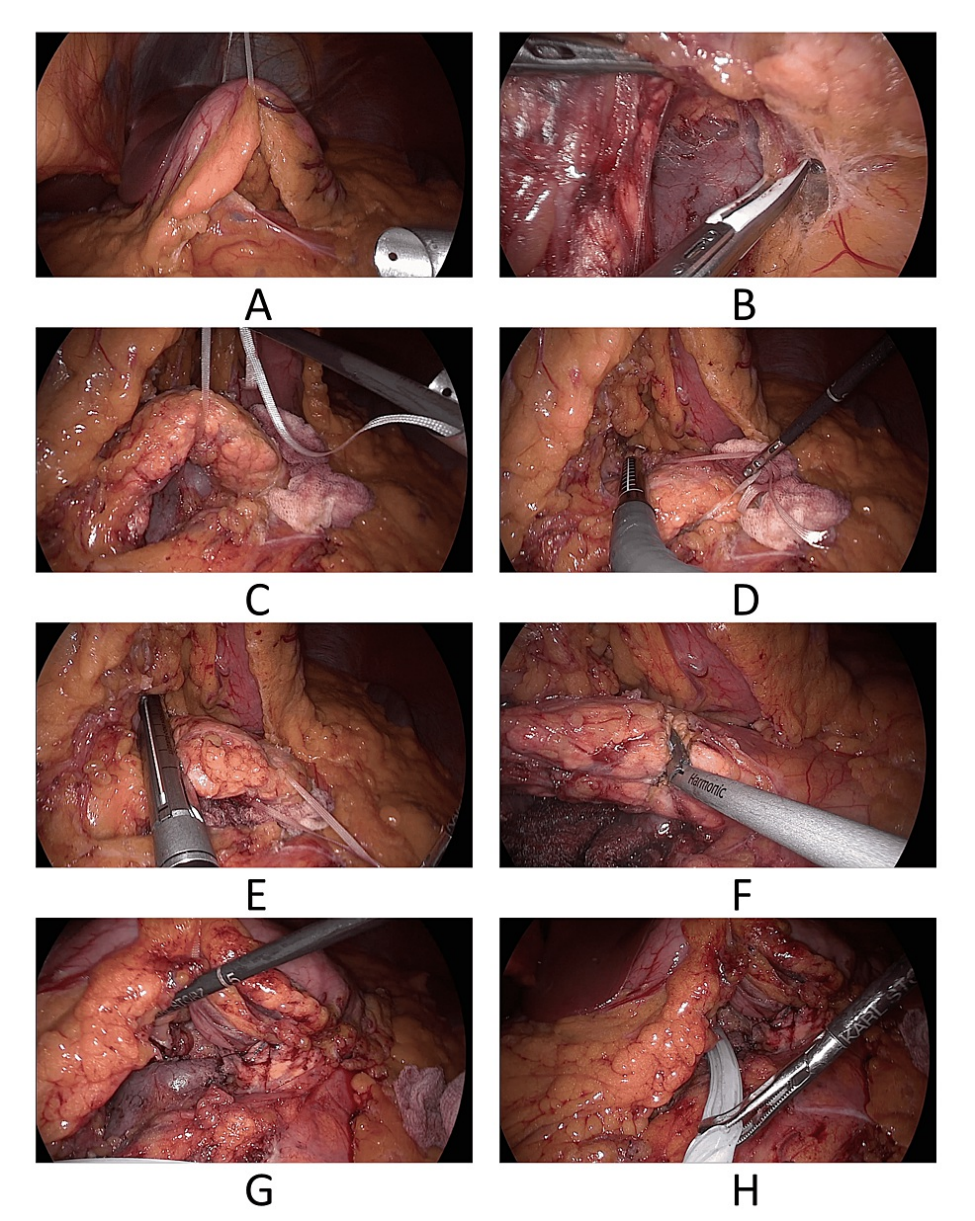
Stages of the procedure A: Displacement of the stomach. B: Tunnelization. C: Mobilization of the pancreatic neck and proximal body. D: Intraoperative ultrasound. E: Proximal transection. F: Distal transection. G: Pancreaticogastrostomy. H: Final view.

One 10-mm supraumbilical trocar, two 5-mm trocars in the right subcostal, and 5- and 12-mm trocars in the left hypochondrium were placed. After accessing the lesser sac, a lesion was found in the body of the pancreas (Figure 1). The stomach was displaced upward. The inferior border of the pancreas was dissected using a LigaSure® device (Covidien, Mansfield, MA, USA), and the superior mesenteric vein was identified under the pancreatic neck. The tunnel was created behind the body, and a splenic vein was exposed. The upper border of the pancreas was dissected, isolating common hepatic and gastroduodenal arteries and identifying the portal vein. Intraoperative ultrasound was utilized to determine the lateral borders of the lesion. The pancreas was transected close to the head by an Echelon-60® linear stapler (Ethicon Endo-Surgery Inc., Johnson & Johnson, Cincinnati, OH, USA) using a golden cartridge. Distally to the tumor, the pancreas was transected by ultrasonic shares, preserving splenic vessels. The specimen was placed in the bag and retrieved through the enlarged 12-mm trocar incision in the left hypochondrium. The lesion was sent for express histology, and the abdomen was deflated. After confirming the diagnosis, invaginated pancreaticogastrostomy was created on the back wall of the stomach. After the checkup of hemostasis, the Blake® drain (Ethicon Inc., Johnson & Johnson, Somerville, NJ, USA) was placed under the anastomosis. The operating time was 315 minutes. Blood loss was 55 mL.

After the surgery, the patient was placed in the ICU. Noninvasive monitoring of vitals and laboratory control of homeostasis parameters were continued. An epidural catheter was used for pain control and stimulation of bowel peristalsis. On postoperative day (POD) 2, the patient was transferred to the ward, and walking was allowed. On POD 4, urinary and epidural catheters were removed, and bowel movements occurred. A nasogastric tube was removed on POD 5, and a liquid diet was started. The patient was discharged on POD 7. No hypoglycemia was noted in the postsurgical period.

After one and four months following the surgery, the patient did well, without any clinical and laboratory evidence of diabetes or malnutrition.

## Discussion

The first central pancreatectomy was done by Guillemin and Bessot in 1957 [1]. It is the parenchyma-sparing technique mostly used for benign and low-grade malignant lesions of the proximal body as a better alternative to the distal (left) pancreatectomy. In medical literature, it is also called middle segment pancreatectomy, segmental pancreatectomy, and medial or median pancreatectomy. The advantage of this method is less postoperative exocrine and endocrine insufficiency and low postoperative morbidity compared to distal pancreatectomy (Table 1).

**Table 1:** Pros and cons of the central pancreatectomy *This disadvantage can be eliminated by performing pancreaticogastrostomy instead of pancreaticojejunostomy.

Advantage	Disadvantage
Low incidence of exocrine insufficiency	Having two pancreatic stumps increases the risk of fistula
Virtually no risk of de novo diabetes development	Pancreaticogastrostomy or pancreaticojejunostomy carries the risk of leakage
Possibility to perform a second-look laparoscopy after surgery	Roux limb creation needs additional anastomosis - jejunojejunostomy*

Laparoscopic pancreatic surgery is superior to the open approach for less postoperative pain, improved cosmesis, quicker recovery, and better results. The morbidity of the open central pancreatectomy is 43.2%, and the mean mortality is 0.24%, while the laparoscopic or robotic technique is associated with a morbidity of 37.3% and a mean of 0% [2]. The possibility of performing a second look into the abdominal cavity is considered to be an advantage of laparoscopic pancreatectomy [3].

Despite the rapid development of laparoscopic pancreas surgery, tumor enucleation, distal pancreatectomy, and pancreatoduodenectomy are the techniques used commonly [4]. Distal pancreatectomy is associated with significant exocrine (5%) and endocrine (4%) insufficiency compared with central pancreatectomy (15.6% and 38%, respectively) [5].

Tumor enucleation is a procedure with excellent parenchyma-sparing ability. However, it is quite difficult to guarantee free margins after it. Bleeding control may be quite challenging. Besides, enucleation carries up to 38% risk of leakage from exposed pancreatic ducts of parenchyma [6].

Laparoscopic central pancreatectomy is performed relatively rarely [4]. The reason is the necessity of pancreatic anastomosis creation, which increases the technical complexity of the procedure and carries the risk of leakage from the proximal or distal stump or both, potentially increasing postsurgical morbidity and mortality [2,7]. The transection of the affected part of the pancreas is not difficult, but creating the anastomosis requires good dexterity. Leakage after central pancreatectomy was reported in as high as 22%-33% of patients, but the source of the fistula was a cephalic stump in all cases [8,9]. A multi-institutional retrospective study confirmed that central pancreatectomy preserves long-term endocrine function [10].

Pancreaticojejunostomy is the most common technique of reconstruction employed, and typically, it is performed with a Roux-en-Y jejunal loop. Leakage and pancreatic fistula incidence after central pancreatectomy followed by pancreaticojejunostomy is 10.6% [11]. Mason analyzed 733 cases of pancreaticogastrostomy after pancreatoduodenectomy [12]. The incidence of leakage for both reconstruction types is similar [10], and the aggregate leakage rate was as low as 4% [12]. The better results of pancreaticogastrostomy could be caused by several factors: the proximity of the stomach to the pancreas allows to minimize tension; the stomach has an excellent blood supply; gastric acid may prevent the activation of pancreatic juice; gastric wall thickness provides better suture-holding capacity than the jejunum; pancreatoduodenectomy needs the creation of extra anastomosis - jejunojejunostomy; pancreaticogastrostomy excludes the development of the Petersen’s hernia [13]. Fixation of the gastric wall on the pancreatic stump can affect gastric motility and delay gastric emptying [14]. However, this suggestion is not confirmed in practice [15].

Two types of pancreaticogastrostomy are being done: duct-to-mucosa and invagination of the pancreatic stump into the stomach.

## Conclusions

Laparoscopic central pancreatectomy for the cystadenoma of the proximal pancreatic body is a feasible method and a better alternative than distal pancreatectomy. Despite the technical complexity, it is safe and avoids the unnecessary removal of the normal pancreatic parenchyma, preserving its exocrine and endocrine function.

Performing this procedure by laparoscopic technique requires mastery and skills, but quick recovery from surgery and less postoperative pain compensate for this practical drawback. Creating pancreaticogastrostomy instead of pancreaticojejunostomy allows to make a single anastomosis and potentially decreases the likelihood of leakage.

## References

[1] GU P, BE M (1957). [Chronic calcifying pancreatitis in renal tuberculosis: pancreatojejunostomy using an original technic]. Mem Acad Chir (Paris).

[2] Hamad A, Novak S, Hogg ME (2017). Robotic central pancreatectomy. J Vis Surg.

[3] Cioltean CL, Bartoş A, Raluca S, Iancu I, Breazu C, Iancu C, Bartoş D (2020). Laparoscopic central pancreatectomy with pancreato-gastric anastomosis for pancreatic cystadenoma. Chirurgia (Bucur).

[4] Machado MA, Surjan RC, Epstein MG, Makdissi FF (2013). Laparoscopic central pancreatectomy: a review of 51 cases. Surg Laparosc Endosc Percutan Tech.

[5] Crippa S, Bassi C, Warshaw AL (2007). Middle pancreatectomy: indications, short- and long-term operative outcomes. Ann Surg.

[6] Fernández-Cruz L, Molina V, Vallejos R, Jiménez Chavarria E, López-Boado MA, Ferrer J (2012). Outcome after laparoscopic enucleation for non-functional neuroendocrine pancreatic tumours. HPB (Oxford).

[7] Winer J, Can MF, Bartlett DL, Zeh HJ, Zureikat AH (2012). The current state of robotic-assisted pancreatic surgery. Nat Rev Gastroenterol Hepatol.

[8] Rotellar F, Pardo F, Montiel C (2008). Totally laparoscopic Roux-en-Y duct-to-mucosa pancreaticojejunostomy after middle pancreatectomy: a consecutive nine-case series at a single institution. Ann Surg.

[9] Sa Cunha A, Rault A, Beau C, Collet D, Masson B (2007). Laparoscopic central pancreatectomy: single institution experience of 6 patients. Surgery.

[10] Sauvanet A, Partensky C, Sastre B (2002). Medial pancreatectomy: a multi-institutional retrospective study of 53 patients by the French Pancreas Club. Surgery.

[11] Sperti C, Pasquali C, Ferronato A, Pedrazzoli S (2000). Median pancreatectomy for tumors of the neck and body of the pancreas. J Am Coll Surg.

[12] Mason GR (1999). Pancreatogastrostomy as reconstruction for pancreatoduodenectomy: review. World J Surg.

[13] Takano S, Ito Y, Watanabe Y, Yokoyama T, Kubota N, Iwai S (2000). Pancreaticojejunostomy versus pancreaticogastrostomy in reconstruction following pancreaticoduodenectomy. Br J Surg.

[14] Yeo CJ, Cameron JL, Maher MM (1995). A prospective randomized trial of pancreaticogastrostomy versus pancreaticojejunostomy after pancreaticoduodenectomy. Ann Surg.

[15] Sugiyama M, Abe N, Ueki H, Masaki T, Mori T, Atomi Y (2004). Pancreaticogastrostomy for reconstruction after medial pancreatectomy. J Am Coll Surg.

